# Everybody's Page

**Published:** 1889-03-16

**Authors:** 


### Everybody's Page.

An old doctor says that the first three months he was in practice he had only one case in his surgery—and that was a case of instruments.

The temperature of our food and drink should be as nearly as possible the same as that of the blood. Iced water and ice-cream are injurious to the stomach, and very hot tea or coffee, especially if swallowed quickly, is equally injurious.

In connexion with the Society of German Naturalists and Physicians, there exists an "International Society for Preventing the Defilement of Rivers," which has as secondary objects the preservation of the purity of the soil and air.

A French chemist has invented a means of freezing milk, so that it can be kept unchanged for weeks together, and be transported any distance in a solid state. When thawed it loses none of its natural qualities.

A Russian surgeon, Dr. Obalinski, recommends the local use of cocaine, simultaneously with the inhalation of chloroform, as a method of producing anæsthesia. By this plan less chloroform is required, and there is less liability to sickness after the return to consciousness.

In the Italian army the number of suicides between the years 1874 and 1880 rose from 40 to 110, or from 20 to 52 in every 100,000 soldiers. Between 1881 and 1886 the number decreased to 50, or about 24 in the 100,000. The proportion of suicides in the army to those in civil life decreased by one-half during the latter period, though they are still twice as numerous.

It is well known that some patients become so much attached to the hospital wards that they would gladly spend their life—a good long life, too—there. For chronic cases of this kind there is kept at the New York Hospital a medicine, bearing in presciptions the name of *Mistura Errabundi*, but known in the vernacular as "The Undertaker's Revenge." It consists of an appetising mixture of copaiba, castor-oil, and quinine, and has been found most efficacious.

Medical men in general are probably not aware that in France, at least, the doctor's claim on the estate of a deceased patient has precedence of all others. Even the landlord's claim for arrears of rent must yield to the doctor's fee. The courts of Rouen, Poictiers, and the Seine have alike decided that as it is an imperative right of humanity that the dying should have the necessary care and treatment, such attendance should be paid for before all other debts.

M. Ungerer believes that flowers and the perfumes distilled from them have a salutary influence on the constitution, and, indeed, may be regarded as a therapeutic agency of high value. He says that residence in a perfumed atmosphere forms a protection from pulmonary affections and arrests the development of phthisis. He says that in the city of La Grasse, where the making of perfumes is largely carried on, phthsis is rare, thanks to the odorous vapours exhaled from the numerous distilleries.

A new acid has been discovered by Dr. D. Hooper which deprives the tongue and palate of the power of tasting sweet and bitter substances for some time after it has been taken. It is derived from an Indian plant, *gymnema sylvestra*, and is therefore called gymnemic acid. If a few drops of the acid, diluted with water, be swallowed, and gingerbread eaten two or three hours afterwards, only the taste of ginger will be recognised. On the other hand, sulphate of quinine taken after the acid, tastes only like so much chalk. Therefore the new acid may become valuable in disguising the taste of nauseous drugs.

Dr. Habershon recommends hypodermic injections of one-sixtieth to one-thirtieth of a grain of strychnia in cases of exhaustion of the heart accompanied by nervous exhaustion.

A New York oculist has discovered a disease of the eye which he traces directly to cigarette smoking. The "cigarette eye" is characterized by dimness, and a film-like gathering over its surface.

Here is a sad warning to the impatient :—

"Here lies the body of Mary Ann Lowder,
Who died of drinking a seidlitz powder ;
She entered into her heavenly rest
Through drinking it before it effervesced."

Sir Francis Drake has been accredited with a pharmaceutical pun. When the ships of the Armada turned and fled before him, he sent to Queen Elizabeth a despatch consisting of the one word "Cantharides," which is, being interpreted, "The Spanish fly."

Once upon a time two farmers quarrelled. Farmer Number One was of an ingeniously spiteful turn, so he bought ten pounds of Epsom salts, and threw it into the well of Farmer Number Two. What was the result? Why, as soon as the water began to taste the report went out that it was a mineral well, and a company was formed which paid Farmer Number Two a handsome sum for his farm, and proceeded to erect a hydropathic.

Some Norwegian shipowners have hit upon a new method of disposing of old ships. To break them up is a laborious and costly process; but the ingenious Norwegians tow them over to the west of Jutland, where trees are scanty and firewood scarce. The hulks are loosely anchored some distance from the shore, and during the first gale the sea will break them up and carry the timber ashore. It is then collected and fetches good prices.

Otto von Guericke, of Magdeburg, was the inventor of cupping glasses. Cupping has of recent years fallen into disrepute, but an American doctor, Dr. George White, of Chicago, has revived it, and claims the best results from it in practice. He offers no special theory of its action, and says that, in some cases, the result seems to be attained by a mental process—like the negro's cure for toothache: put some red pepper in your eye, and you will think so much about your eye that you will forget your tooth.

Here is a testimony to the value of fat :—On the 14th of December, 1810, a pig was buried in its sty through the fall of part of the cliff under Dover Castle. The sty consisted of a cave in the rock about six feet square, and boarded in front; and when the accident happened the pig was in good condition, weighing about 160 lbs. Five months afterwards, on the 23rd of May, 1811, some workmen who were engaged in clearing away the débris of the fallen cliff, mentioned to Dr. Mantell, a well-known geologist of the day, and a Fellow of the Linnean Society, that they were sure they heard the pig whining. He thought the statement incredible, but ordered them to clear away the chalk as fast as they could; and, sure enough, when they got to the sty, the pig was there, weak and emaciated, fallen to only a fourth of his former weight, but alive. In 160 days he had been strictly self-supporting, living on the store of fat he had laid up in more prosperous times. There were, however, evidences of his sufferings in the wood that shut in the sty being nibbled away in places, while he had licked the sides of the cave smooth in his attempt to obtain the moisture exuding from the rock.

